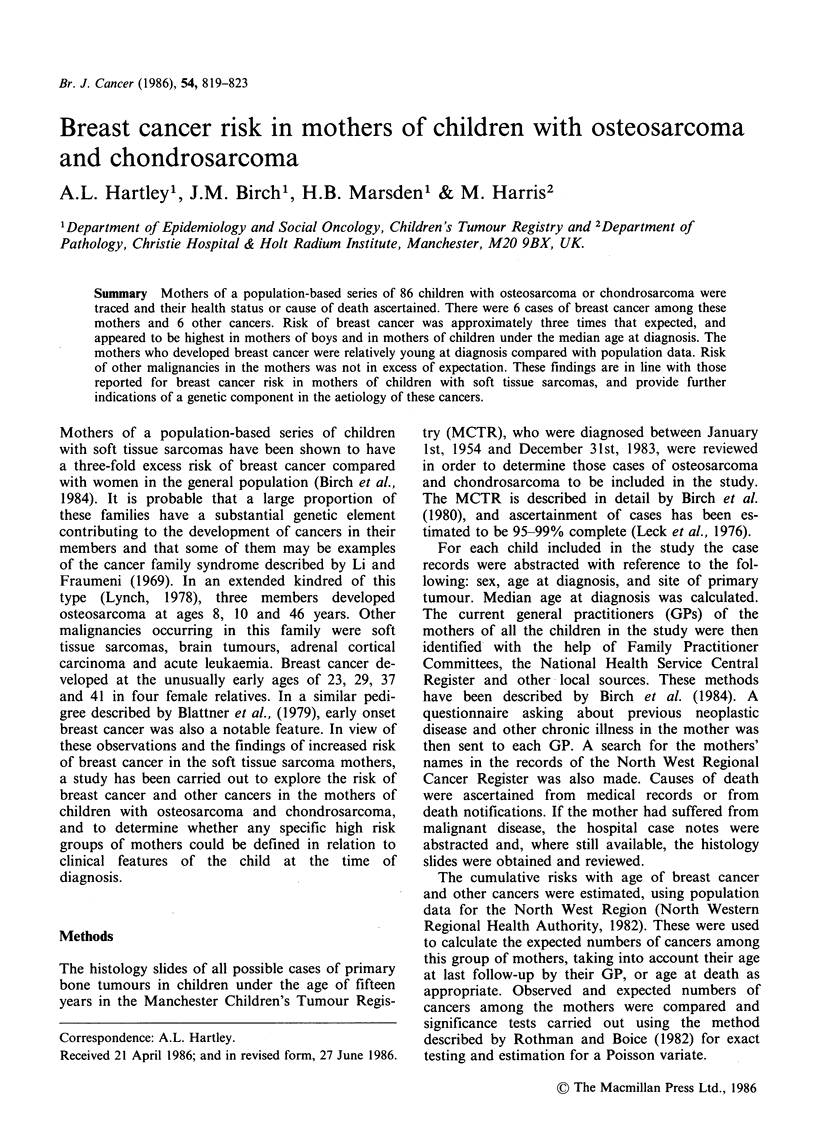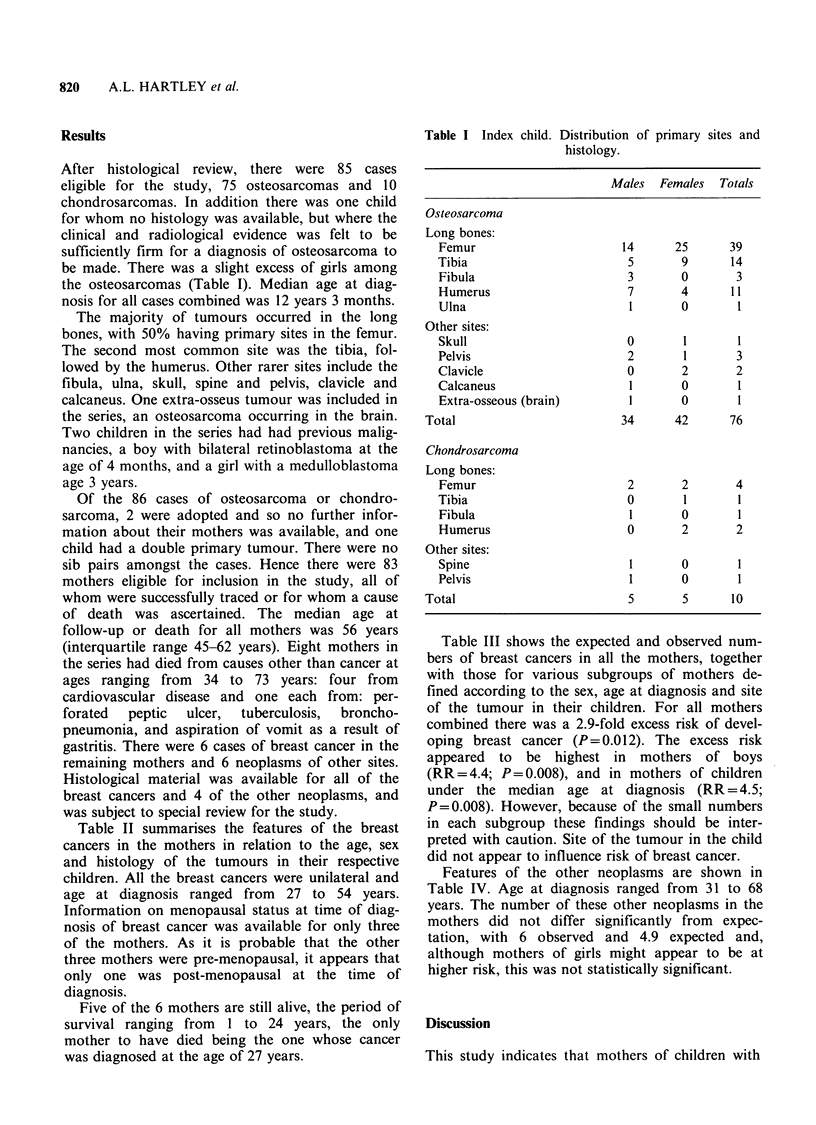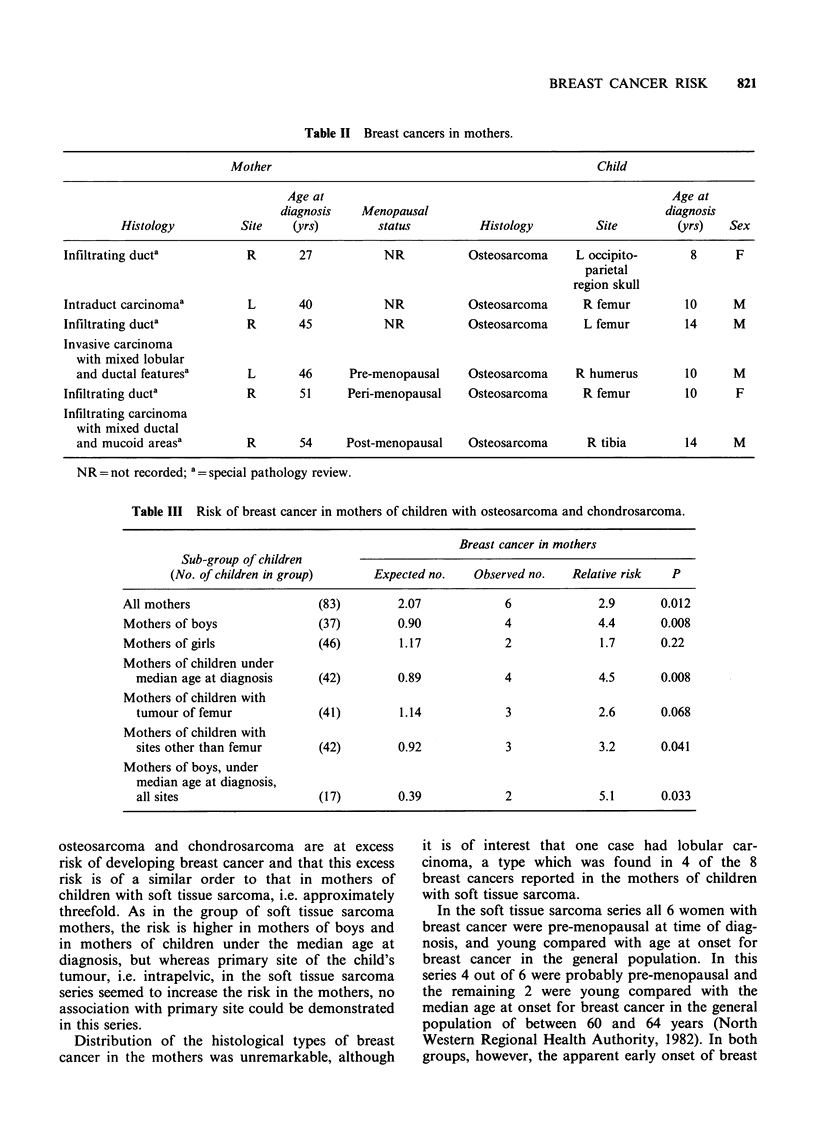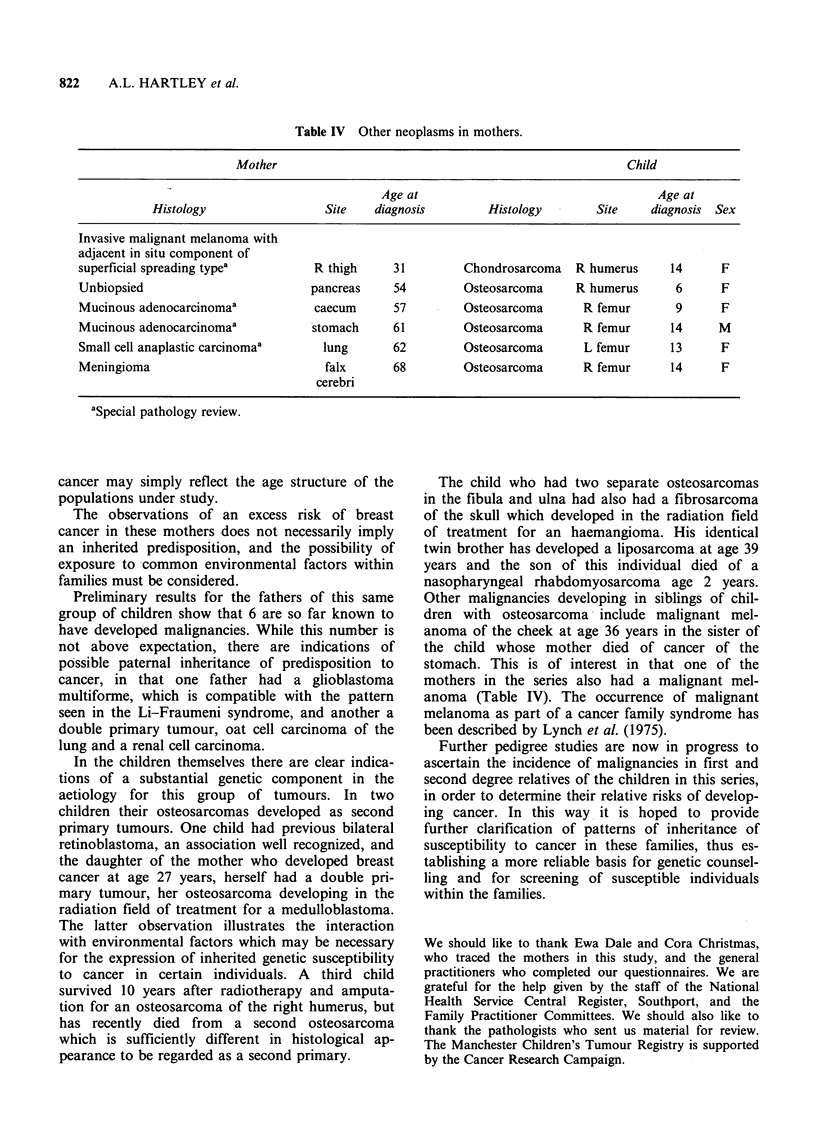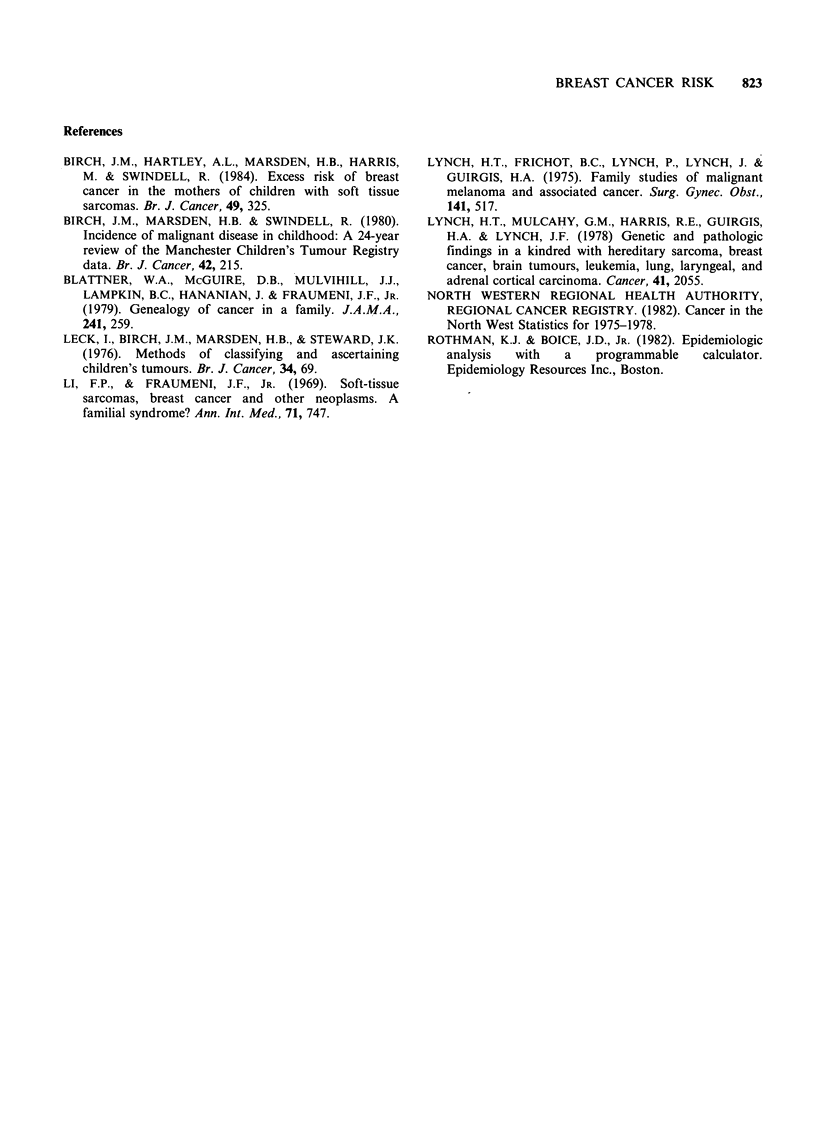# Breast cancer risk in mothers of children with osteosarcoma and chondrosarcoma.

**DOI:** 10.1038/bjc.1986.245

**Published:** 1986-11

**Authors:** A. L. Hartley, J. M. Birch, H. B. Marsden, M. Harris

## Abstract

Mothers of a population-based series of 86 children with osteosarcoma or chondrosarcoma were traced and their health status or cause of death ascertained. There were 6 cases of breast cancer among these mothers and 6 other cancers. Risk of breast cancer was approximately three times that expected, and appeared to be highest in mothers of boys and in mothers of children under the median age at diagnosis. The mothers who developed breast cancer were relatively young at diagnosis compared with population data. Risk of other malignancies in the mothers was not in excess of expectation. These findings are in line with those reported for breast cancer risk in mothers of children with soft tissue sarcomas, and provide further indications of a genetic component in the aetiology of these cancers.


*Br. J. Cancer* (1986), **54**, 819–823

### Breast cancer risk in mothers of children with osteosarcoma and chondrosarcoma

A.L. Hartley[1], J.M. Birch[1], H.B. Marsden[1] & M. Harris[2]

[1]*Department of Epidemiology and Social Oncology, Children's Tumour Registry and* [2]*Department of Pathology, Christie Hospital & Holt Radium Institute, Manchester, M20 9BX, UK.*

**Summary** Mothers of a population-based series of 86 children with osteosarcoma or chondrosarcoma were traced and their health status or cause of death ascertained. There were 6 cases of breast cancer among these mothers and 6 other cancers. Risk of breast cancer was approximately three times that expected, and appeared to be highest in mothers of boys and in mothers of children under the median age at diagnosis. The mothers who developed breast cancer were relatively young at diagnosis compared with population data. Risk of other malignancies in the mothers was not in excess of expectation. These findings are in line with those reported for breast cancer risk in mothers of children with soft tissue sarcomas, and provide further indications of a genetic component in the aetiology of these cancers.

Mothers of a population-based series of children with soft tissue sarcomas have been shown to have a three-fold excess risk of breast cancer compared with women in the general population (Birch *et al.*, 1984). It is probable that a large proportion of these families have a substantial genetic element contributing to the development of cancers in their members and that some of them may be examples of the cancer family syndrome described by Li and Fraumeni (1969). In an extended kindred of this type (Lynch, 1978), three members developed osteosarcoma at ages 8, 10 and 46 years. Other malignancies occurring in this family were soft tissue sarcomas, brain tumours, adrenal cortical carcinoma and acute leukaemia. Breast cancer developed at the unusually early ages of 23, 29, 37 and 41 in four female relatives. In a similar pedigree described by Blattner *et al.*, (1979), early onset breast cancer was also a notable feature. In view of these observations and the findings of increased risk of breast cancer in the soft tissue sarcoma mothers, a study has been carried out to explore the risk of breast cancer and other cancers in the mothers of children with osteosarcoma and chondrosarcoma, and to determine whether any specific high risk groups of mothers could be defined in relation to clinical features of the child at the time of diagnosis.

### Methods

The histology slides of all possible cases of primary bone tumours in children under the age of fifteen years in the Manchester Children's Tumour Registry (MCTR), who were diagnosed between January 1st, 1954 and December 31st, 1983, were reviewed in order to determine those cases of osteosarcoma and chondrosarcoma to be included in the study. The MCTR is described in detail by Birch *et al.* (1980), and ascertainment of cases has been estimated to be 95–99% complete (Leck *et al.*, 1976).

For each child included in the study the case records were abstracted with reference to the following: sex, age at diagnosis, and site of primary tumour. Median age at diagnosis was calculated. The current general practitioners (GPs) of the mothers of all the children in the study were then identified with the help of Family Practitioner Committees, the National Health Service Central Register and other local sources. These methods have been described by Birch *et al.* (1984). A questionnaire asking about previous neoplastic disease and other chronic illness in the mother was then sent to each GP. A search for the mothers' names in the records of the North West Regional Cancer Register was also made. Causes of death were ascertained from medical records or from death notifications. If the mother had suffered from malignant disease, the hospital case notes were abstracted and, where still available, the histology slides were obtained and reviewed.

The cumulative risks with age of breast cancer and other cancers were estimated, using population data for the North West Region (North Western Regional Health Authority, 1982). These were used to calculate the expected numbers of cancers among this group of mothers, taking into account their age at last follow-up by their GP, or age at death as appropriate. Observed and expected numbers of cancers among the mothers were compared and significance tests carried out using the method described by Rothman and Boice (1982) for exact testing and estimation for a Poisson variate.

Correspondence: A.L. Hartley.

Received 21 April 1986; and in revised form, 27 June 1986.

© The Macmillan Press Ltd., 1986

### Results

After histological review, there were 85 cases eligible for the study, 75 osteosarcomas and 10 chondrosarcomas. In addition there was one child for whom no histology was available, but where the clinical and radiological evidence was felt to be sufficiently firm for a diagnosis of osteosarcoma to be made. There was a slight excess of girls among the osteosarcomas (Table I). Median age at diagnosis for all cases combined was 12 years 3 months.

The majority of tumours occurred in the long bones, with 50% having primary sites in the femur. The second most common site was the tibia, followed by the humerus. Other rarer sites include the fibula, ulna, skull, spine and pelvis, clavicle and calcaneus. One extra-osseus tumour was included in the series, an osteosarcoma occurring in the brain. Two children in the series had had previous malignancies, a boy with bilateral retinoblastoma at the age of 4 months, and a girl with a medulloblastoma age 3 years.

Of the 86 cases of osteosarcoma or chondrosarcoma, 2 were adopted and so no further information about their mothers was available, and one child had a double primary tumour. There were no sib pairs amongst the cases. Hence there were 83 mothers eligible for inclusion in the study, all of whom were successfully traced or for whom a cause of death was ascertained. The median age at follow-up or death for all mothers was 56 years (interquartile range 45–62 years). Eight mothers in the series had died from causes other than cancer at ages ranging from 34 to 73 years: four from cardiovascular disease and one each from: perforated peptic ulcer, tuberculosis, bronchopneumonia, and aspiration of vomit as a result of gastritis. There were 6 cases of breast cancer in the remaining mothers and 6 neoplasms of other sites. Histological material was available for all of the breast cancers and 4 of the other neoplasms, and was subject to special review for the study.

Table II summarises the features of the breast cancers in the mothers in relation to the age, sex and histology of the tumours in their respective children. All the breast cancers were unilateral and age at diagnosis ranged from 27 to 54 years. Information on menopausal status at time of diagnosis of breast cancer was available for only three of the mothers. As it is probable that the other three mothers were pre-menopausal, it appears that only one was post-menopausal at the time of diagnosis.

Five of the 6 mothers are still alive, the period of survival ranging from 1 to 24 years, the only mother to have died being the one whose cancer was diagnosed at the age of 27 years.

**Table I** Index child. Distribution of primary sites and histology.

| | *Males* | *Females* | *Totals* |
|---|---|---|---|
| *Osteosarcoma* | | | |
| Long bones: | | | |
| Femur | 14 | 25 | 39 |
| Tibia | 5 | 9 | 14 |
| Fibula | 3 | 0 | 3 |
| Humerus | 7 | 4 | 11 |
| Ulna | 1 | 0 | 1 |
| Other sites: | | | |
| Skull | 0 | 1 | 1 |
| Pelvis | 2 | 1 | 3 |
| Clavicle | 0 | 2 | 2 |
| Calcaneus | 1 | 0 | 1 |
| Extra-osseous (brain) | 1 | 0 | 1 |
| Total | 34 | 42 | 76 |
| *Chondrosarcoma* | | | |
| Long bones: | | | |
| Femur | 2 | 2 | 4 |
| Tibia | 0 | 1 | 1 |
| Fibula | 1 | 0 | 1 |
| Humerus | 0 | 2 | 2 |
| Other sites: | | | |
| Spine | 1 | 0 | 1 |
| Pelvis | 1 | 0 | 1 |
| Total | 5 | 5 | 10 |

Table III shows the expected and observed numbers of breast cancers in all the mothers, together with those for various subgroups of mothers defined according to the sex, age at diagnosis and site of the tumour in their children. For all mothers combined there was a 2.9-fold excess risk of developing breast cancer ($P=0.012$). The excess risk appeared to be highest in mothers of boys (RR=4.4; $P=0.008$), and in mothers of children under the median age at diagnosis (RR=4.5; $P=0.008$). However, because of the small numbers in each subgroup these findings should be interpreted with caution. Site of the tumour in the child did not appear to influence risk of breast cancer.

Features of the other neoplasms are shown in Table IV. Age at diagnosis ranged from 31 to 68 years. The number of these other neoplasms in the mothers did not differ significantly from expectation, with 6 observed and 4.9 expected and, although mothers of girls might appear to be at higher risk, this was not statistically significant.

### Discussion

This study indicates that mothers of children with

**Table II** Breast cancers in mothers.

| Mother | | | | Child | | | |
|---|---|---|---|---|---|---|---|
| *Histology* | *Site* | *Age at diagnosis (yrs)* | *Menopausal status* | *Histology* | *Site* | *Age at diagnosis (yrs)* | *Sex* |
| Infiltrating duct[a] | R | 27 | NR | Osteosarcoma | L occipito-parietal region skull | 8 | F |
| Intraduct carcinoma[a] | L | 40 | NR | Osteosarcoma | R femur | 10 | M |
| Infiltrating duct[a] | R | 45 | NR | Osteosarcoma | L femur | 14 | M |
| Invasive carcinoma with mixed lobular and ductal features[a] | L | 46 | Pre-menopausal | Osteosarcoma | R humerus | 10 | M |
| Infiltrating duct[a] | R | 51 | Peri-menopausal | Osteosarcoma | R femur | 10 | F |
| Infiltrating carcinoma with mixed ductal and mucoid areas[a] | R | 54 | Post-menopausal | Osteosarcoma | R tibia | 14 | M |

NR = not recorded; [a] = special pathology review.

**Table III** Risk of breast cancer in mothers of children with osteosarcoma and chondrosarcoma.

| *Sub-group of children* | *(No. of children in group)* | *Breast cancer in mothers* | | | |
|---|---|---|---|---|---|
| | | *Expected no.* | *Observed no.* | *Relative risk* | *P* |
| All mothers | (83) | 2.07 | 6 | 2.9 | 0.012 |
| Mothers of boys | (37) | 0.90 | 4 | 4.4 | 0.008 |
| Mothers of girls | (46) | 1.17 | 2 | 1.7 | 0.22 |
| Mothers of children under median age at diagnosis | (42) | 0.89 | 4 | 4.5 | 0.008 |
| Mothers of children with tumour of femur | (41) | 1.14 | 3 | 2.6 | 0.068 |
| Mothers of children with sites other than femur | (42) | 0.92 | 3 | 3.2 | 0.041 |
| Mothers of boys, under median age at diagnosis, all sites | (17) | 0.39 | 2 | 5.1 | 0.033 |

osteosarcoma and chondrosarcoma are at excess risk of developing breast cancer and that this excess risk is of a similar order to that in mothers of children with soft tissue sarcoma, i.e. approximately threefold. As in the group of soft tissue sarcoma mothers, the risk is higher in mothers of boys and in mothers of children under the median age at diagnosis, but whereas primary site of the child's tumour, i.e. intrapelvic, in the soft tissue sarcoma series seemed to increase the risk in the mothers, no association with primary site could be demonstrated in this series.

Distribution of the histological types of breast cancer in the mothers was unremarkable, although it is of interest that one case had lobular carcinoma, a type which was found in 4 of the 8 breast cancers reported in the mothers of children with soft tissue sarcoma.

In the soft tissue sarcoma series all 6 women with breast cancer were pre-menopausal at time of diagnosis, and young compared with age at onset for breast cancer in the general population. In this series 4 out of 6 were probably pre-menopausal and the remaining 2 were young compared with the median age at onset for breast cancer in the general population of between 60 and 64 years (North Western Regional Health Authority, 1982). In both groups, however, the apparent early onset of breast

**Table IV** Other neoplasms in mothers.

| *Mother* | | | *Child* | | | |
|---|---|---|---|---|---|---|
| *Histology* | *Site* | *Age at diagnosis* | *Histology* | *Site* | *Age at diagnosis* | *Sex* |
| Invasive malignant melanoma with adjacent in situ component of superficial spreading type[a] | R thigh | 31 | Chondrosarcoma | R humerus | 14 | F |
| Unbiopsied | pancreas | 54 | Osteosarcoma | R humerus | 6 | F |
| Mucinous adenocarcinoma[a] | caecum | 57 | Osteosarcoma | R femur | 9 | F |
| Mucinous adenocarcinoma[a] | stomach | 61 | Osteosarcoma | R femur | 14 | M |
| Small cell anaplastic carcinoma[a] | lung | 62 | Osteosarcoma | L femur | 13 | F |
| Meningioma | falx cerebri | 68 | Osteosarcoma | R femur | 14 | F |

[a]Special pathology review.

cancer may simply reflect the age structure of the populations under study.

The observations of an excess risk of breast cancer in these mothers does not necessarily imply an inherited predisposition, and the possibility of exposure to common environmental factors within families must be considered.

Preliminary results for the fathers of this same group of children show that 6 are so far known to have developed malignancies. While this number is not above expectation, there are indications of possible paternal inheritance of predisposition to cancer, in that one father had a glioblastoma multiforme, which is compatible with the pattern seen in the Li–Fraumeni syndrome, and another a double primary tumour, oat cell carcinoma of the lung and a renal cell carcinoma.

In the children themselves there are clear indications of a substantial genetic component in the aetiology for this group of tumours. In two children their osteosarcomas developed as second primary tumours. One child had previous bilateral retinoblastoma, an association well recognized, and the daughter of the mother who developed breast cancer at age 27 years, herself had a double primary tumour, her osteosarcoma developing in the radiation field of treatment for a medulloblastoma. The latter observation illustrates the interaction with environmental factors which may be necessary for the expression of inherited genetic susceptibility to cancer in certain individuals. A third child survived 10 years after radiotherapy and amputation for an osteosarcoma of the right humerus, but has recently died from a second osteosarcoma which is sufficiently different in histological appearance to be regarded as a second primary.

The child who had two separate osteosarcomas in the fibula and ulna had also had a fibrosarcoma of the skull which developed in the radiation field of treatment for an haemangioma. His identical twin brother has developed a liposarcoma at age 39 years and the son of this individual died of a nasopharyngeal rhabdomyosarcoma age 2 years. Other malignancies developing in siblings of children with osteosarcoma include malignant melanoma of the cheek at age 36 years in the sister of the child whose mother died of cancer of the stomach. This is of interest in that one of the mothers in the series also had a malignant melanoma (Table IV). The occurrence of malignant melanoma as part of a cancer family syndrome has been described by Lynch *et al.* (1975).

Further pedigree studies are now in progress to ascertain the incidence of malignancies in first and second degree relatives of the children in this series, in order to determine their relative risks of developing cancer. In this way it is hoped to provide further clarification of patterns of inheritance of susceptibility to cancer in these families, thus establishing a more reliable basis for genetic counselling and for screening of susceptible individuals within the families.

We should like to thank Ewa Dale and Cora Christmas, who traced the mothers in this study, and the general practitioners who completed our questionnaires. We are grateful for the help given by the staff of the National Health Service Central Register, Southport, and the Family Practitioner Committees. We should also like to thank the pathologists who sent us material for review. The Manchester Children's Tumour Registry is supported by the Cancer Research Campaign.

### References

BIRCH, J.M., HARTLEY, A.L., MARSDEN, H.B., HARRIS, M. & SWINDELL, R. (1984). Excess risk of breast cancer in the mothers of children with soft tissue sarcomas. *Br. J. Cancer*, **49**, 325.

BIRCH, J.M., MARSDEN, H.B. & SWINDELL, R. (1980). Incidence of malignant disease in childhood: A 24-year review of the Manchester Children's Tumour Registry data. *Br. J. Cancer*, **42**, 215.

BLATTNER, W.A., McGUIRE, D.B., MULVIHILL, J.J., LAMPKIN, B.C., HANANIAN, J. & FRAUMENI, J.F., JR. (1979). Genealogy of cancer in a family. *J.A.M.A.*, **241**, 259.

LECK, I., BIRCH, J.M., MARSDEN, H.B., & STEWARD, J.K. (1976). Methods of classifying and ascertaining children's tumours. *Br. J. Cancer*, **34**, 69.

LI, F.P., & FRAUMENI, J.F., JR. (1969). Soft-tissue sarcomas, breast cancer and other neoplasms. A familial syndrome? *Ann. Int. Med.*, **71**, 747.

LYNCH, H.T., FRICHOT, B.C., LYNCH, P., LYNCH, J. & GUIRGIS, H.A. (1975). Family studies of malignant melanoma and associated cancer. *Surg. Gynec. Obst.*, **141**, 517.

LYNCH, H.T., MULCAHY, G.M., HARRIS, R.E., GUIRGIS, H.A. & LYNCH, J.F. (1978) Genetic and pathologic findings in a kindred with hereditary sarcoma, breast cancer, brain tumours, leukemia, lung, laryngeal, and adrenal cortical carcinoma. *Cancer*, **41**, 2055.

NORTH WESTERN REGIONAL HEALTH AUTHORITY, REGIONAL CANCER REGISTRY. (1982). Cancer in the North West Statistics for 1975–1978.

ROTHMAN, K.J. & BOICE, J.D., JR. (1982). Epidemiologic analysis with a programmable calculator. Epidemiology Resources Inc., Boston.